# Pathophysiological roles and therapeutic potential of voltage-gated ion channels (VGICs) in pain associated with herpesvirus infection

**DOI:** 10.1186/s13578-020-00430-2

**Published:** 2020-05-24

**Authors:** Qiaojuan Zhang, Miguel Martin-Caraballo, Shaochung V. Hsia

**Affiliations:** Department of Pharmaceutical Sciences, School of Pharmacy and Health Professions, University of Maryland Eastern Shore, Princess Anne, MD 21853 USA

**Keywords:** Herpesvirus, HSV-1, Latency, Reactivation, Voltage-gated sodium channel, T-type calcium channels, Inflammatory pain, Neuropathic pain

## Abstract

Herpesvirus is ranked as one of the grand old members of all pathogens. Of all the viruses in the superfamily, Herpes simplex virus type 1 (HSV-1) is considered as a model virus for a variety of reasons. In a permissive non-neuronal cell culture, HSV-1 concludes the entire life cycle in approximately 18–20 h, encoding approximately 90 unique transcriptional units. In latency, the robust viral gene expression is suppressed in neurons by a group of noncoding RNA. Historically the lesions caused by the virus can date back to centuries ago. As a neurotropic pathogen, HSV-1 is associated with painful oral lesions, severe keratitis and lethal encephalitis. Transmission of pain signals is dependent on the generation and propagation of action potential in sensory neurons. T-type Ca^2+^ channels serve as a preamplifier of action potential generation. Voltage-gated Na^+^ channels are the main components for action potential production. This review summarizes not only the voltage-gated ion channels in neuropathic disorders but also provides the new insights into HSV-1 induced pain.

## Introduction

Herpes simplex virus type 1 (HSV-1) is a neurotropic pathogen associated with the development of painful oral lesions, severe keratitis, lethal encephalitis as well as Alzheimer’s disease [1–3]. Untreated herpes simplex encephalitis (HSE) causes over 70% cases of death, and at least half of the survivors experienced severe neurological damage [4, 5]. After acute infection of epithelial cells, HSV-1 usually establishes latent infection in dorsal root ganglion (DRG), trigeminal ganglia (TG) or other sensory neurons. Reactivation of HSV-1 from latency occurs in response to various factors, such as hyperthermia, heat shock, explantation, ultraviolet irradiation and skin trauma [6]. HSV-1 entry could occur by either endocytic pathway or fusion of viral glycoproteins with membrane receptors [7]. Once the viral genome enters the nucleoplasm, viral gene expression will start immediately, followed by viral replication and synthesis of viral protein, capsid assembly, DNA packaging and then viral particles assembly and release. The lytic infection of HSV-1 can lead to necrosis or apoptosis of infected cells [8]. Abnormal neuronal cell death in response to HSV-1 infection could cause pain sensations, formication, paresthesia, and even touch loss around initial infection area, which have been reported to be related to altered neuronal excitability [9].

Voltage-gated ion channels, such as voltage-gated sodium channels (VGSCs) and voltage-gated calcium channels (VGCCs), play an essential role in neuronal excitability and pain signaling. Voltage-gated Na^+^ channels are widely expressed in excitable cells, including peripheral neurons, central neurons, cardiac cells and muscle cells. LVA (or T-type) calcium channels are expressed throughout the whole human body, including the nervous system, musculoskeletal system, cardiovascular system and endocrine system.

Transmission of pain information is highly dependent on the generation of action potential which requires activation of voltage-gated Na^+^ channels. In neurons, increased expression of voltage-gated Na^+^ channels indicates increased neuronal excitability, which usually occurs in lowered threshold needed for generation of action potentials and increased spontaneous firing or firing frequency. Increased neuronal excitability results in amplified pain signaling, while decreased neuronal excitability alleviates the transmission of pain signals. HSV-1 lytic infection triggers complete internalization of voltage-activated Na^+^ channel proteins from the plasma membrane of neurons, resulting in subsequent loss of the electrical excitability [10]. However, HSV-1 syncytial strain infection of DRG sensory neurons can trigger the spontaneous firing activity [11]. HSV-1 latent infection increases the expression of voltage-gated Na^+^ channels, which may enhance the pain sensation, while HSV-1 reactivation decreases the voltage-gated Na^+^ channel activity [6, 12, 13].

Voltage-gated Ca^2+^ channels (VGCCs) are classified into low voltage- and high voltage-activated (LVA and HVA) Ca^2+^ channels based on their biophysical and pharmacological properties. Improper regulation of LVA (or T-type) Ca^2+^ channels has been implicated in several forms of diseases, including pain, epilepsy [14], cancer [15], as well as Parkinson’s disease [16]. HSV-1 lytic infection eliminates the excitability of dorsal root ganglion (DRG) neurons [10, 12, 17, 18]. T-type Ca^2+^ channel activation could elevate the intracellular Ca^2+^ concentration and increase the neuronal excitability, which is known to affect the transmission of pain information [19].

## HSV-1 and pain

The mechanism of nociceptive pain, inflammatory pain and neuropathic pain is fundamentally different, though, etiologies sometimes are very similar. Nociceptive pain is induced by acute high intensity noxious stimuli, and can be resolved after removal of the stimuli [20, 21]. Tissue damage causes the release of inflammatory mediators, such as serotonin, bradykinin, histamine, prostaglandins, leukotrienes as well as potassium ions. These factors are reported to induce inflammatory pain by stimulating nociceptors located at the nerve ending or neuronal cell bodies [22–24]. These compounds also contribute to peripheral sensitization and central sensitization by activating protein kinase A (PKA) and protein kinase C (PKC) second messengers, resulting in reduced action potential threshold and increased responsiveness [24–26]. Neuropathic pain is the chronic pain associated with nerve injury or dysfunction. The recovery of nerve injury could not obscure the pain sensation. Ectopic discharges and spontaneous firing activity were developed in the injured afferent neurons [26, 27], while the peripheral sensitization was developed in uninjured afferent neurons [26, 28]. The central sensitization was also developed during neuropathic pain through prolonged nociceptive input [29]. Allodynia is a painful sensation to non-painful normal stimuli, and hyperalgesia is an abnormally increased pain sensation to a painful stimulus. Pain sensitization induced allodynia and hyperalgesia resulted from inflammatory pain or neuropathic pain could be also induced by HSV-1 infection.

**Fig. 1:**
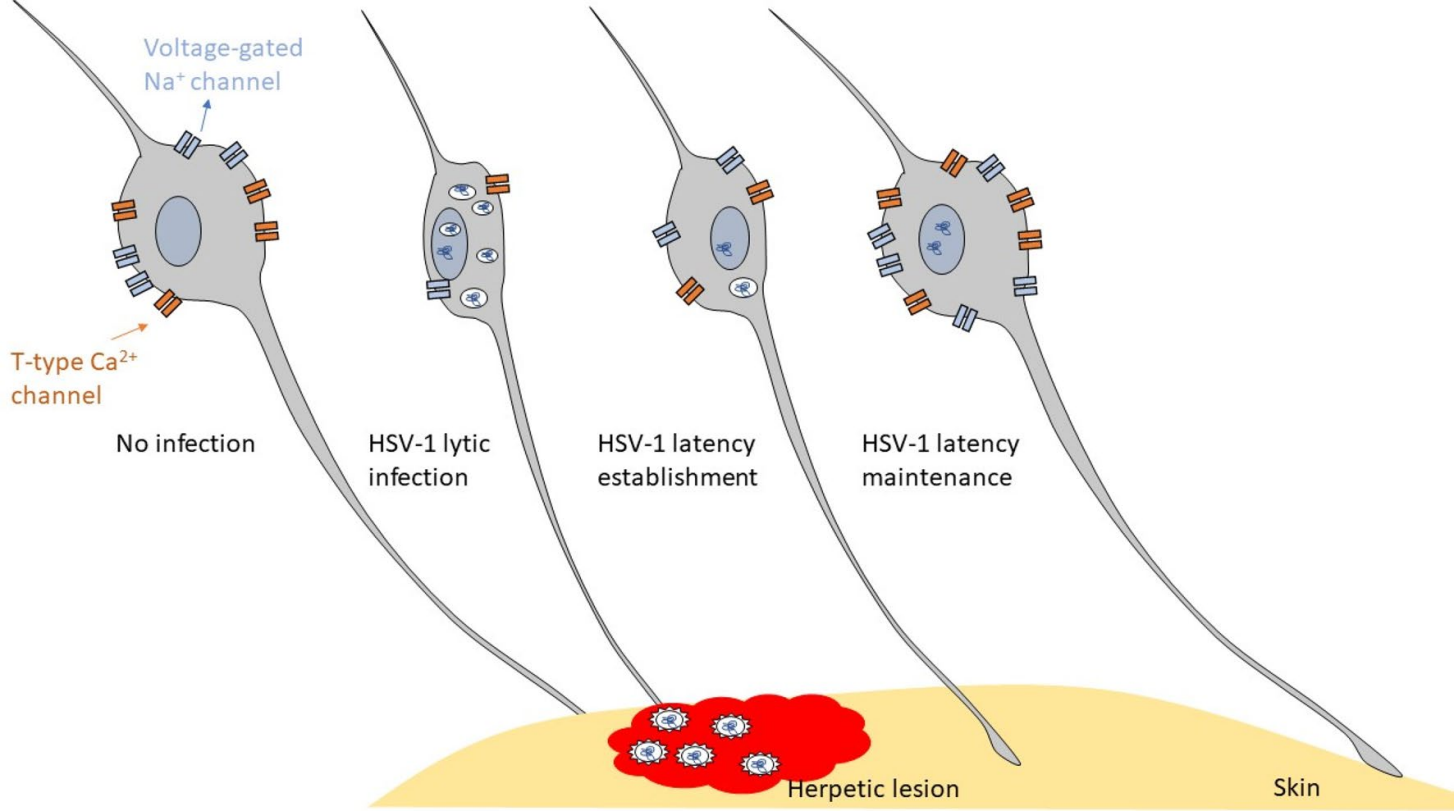
The voltage-gated Na^+^ channel and T-type Ca^2+^ channel expression of sensory neurons in different stages of HSV-1 infection. Most of the sensory neurons express abundant voltage-gated Na^+^ channels and T-type Ca^2+^ channels. HSV-1 lytic infection of sensory neurons induces a significant decrease of these channels, HSV-1 latency establishment decrease these channels, HSV-1 latency maintenance restore theses channels or even increase these channels

HSV-1 lytic infection or reactivation causes tissue damage that can result in inflammatory pain, and the treatment for viral active infection is cost-effective. Acyclovir (ACV) has been applied on herpesvirus infection for decades, especially on HSV-1 infection. In viral infection induced tissue damage, ACV inhibits viral replication and release, which helps to heal the damaged tissue and remove the inflammatory pain. However, in HSV-1 infection induced neuropathy, ACV treatment could not completely repair nerve damage induced neuropathic pain. Although neuropathic pain was rarely reported in HSV related disease [most commonly, in other alpha herpesviruses infection, such as varicella zoster virus (VZV) or pseudorabies virus (PRV)], more and more reports showed that HSV-1/2 could induce long-lasting pain sensation without active HSV-1/2 infection. Clinical case reports indicate that HSV-1 could induce post-hepatic neuralgia, such as long lasting oral-facial burning pain and hypersensitivity or chronic occipital neuralgia [30, 31]. HSV-2 infection was also reported to be associated with hyperalgesia, unilateral or bilateral chronic neuropathic pain in the sacral area [32, 33]. Pain signal is transmitted through action potential generation and propagation. Voltage-gated Na^+^ channels and T-type Ca^2+^ channels are the two main contributors of action potential that are recognized to play profound roles in inflammatory and neuropathic pain. The possible regulatory effects of HSV-1 infection on sensory neurons are based on the stages of viral infection (Fig. 1). Comprehensive characterization of the underlying mechanism of HSV-1 infection induced abnormal pain sensation or neuropathic pain will lay the foundation to treat HSV-1 associated herpetic pain.

### Voltage-gated Na^+^ channels and pain

HSV-1 can latently infect the dorsal root ganglia (DRG) and trigeminal ganglia (TG) neurons. DRG neurons express a variety of ion channels and receptors, including voltage-gated Na^+^ channels and Ca^2+^ channels, ATP-sensitive receptors, NMDA receptors and AMPA receptors. These channels and receptors mediate the transduction, conduction and synaptic transmission processes required for the propagation of sensory information. Neurite degeneration or axon demyelination caused by nerve injury may trigger remodeling of ion channels and receptors, which can increase neuronal excitability and change the firing properties of these neurons [34–36]. The increased neuronal excitability largely results from a switch in the subtype of voltage-gated Na^+^ channels, increased trafficking or altered kinetic properties of these channels [36–38].

Ten subtypes of voltage-gated Na^+^ channels have been identified in mammals, i.e. Nav1.1 through Nav1.9 and NaX. Most of these subtypes are sensitive to nanomolar concentration of the puffer fish-derived toxin tetrodotoxin (TTX), named TTX-sensitive channels (Nav1.1, Nav1.2, Nav1.3, Nav1.4, Nav1.6 and Nav1.7). These channels show fast activating and inactivating current property. The rest of Na^+^ channels generate TTX-resistant sodium currents, which shows slow activating and inactivating kinetics [38]. In primary sensory neurons or DRG neurons, voltage-gated Na^+^ channel expression can be induced by dibutyryl-cyclic adenosine monophosphate (db-AMP), forskolin and diacylglycerol (DAG) through activation of protein kinase A (PKA) or protein kinase C (PKC) signaling [6, 12, 39]. Many neurotrophies, such as nerve growth factor (NGF), brain-derived neurotrophic factors (BDNF), glial-derived neurotrophic factor (GDNF) as well as neurotrophin-3 (NT-3), are reported to modulate the expression of voltage-gated Na^+^ channels. Therefore, in many in vitro models, neuronal cell differentiation can be triggered by these factors [6, 12, 13].

Voltage-gated Na^+^ channels are widely expressed in both the peripheral and central nerve systems, while Nav1.4 is only expressed in skeletal muscle and Nav1.5 is mainly expressed in cardiac cells [36, 40]. These channels are usually expressed in excitable cells and participate in generating the action potentials. Interestingly, voltage-gated Na^+^ channels are also expressed in non-excitable cells, such as cancer cells, where they are mainly involved in cancer cell proliferation, migration and invasion [41].

Functional expression of voltage-gated Na^**+**^ channels in neurons participates in action potential generation, which is required for transmission of pain information. Nav1.1 or Nav1.7 channel transmit noxious mechanical stimuli through Aδ sensory fibers, while Nav1.8 and Nav1.9 mainly transmit noxious thermal stimuli through C fibers [42]. In injured nerve models, the abnormal expression of sodium channels leads to the development of inflammatory or neuropathic pain, such as hyperalgesia or allodynia. Understanding the mechanism of hyperalgesia and allodynia after nerve injury or neuropathic disorders that involves alteration in voltage-gated Na^+^ channels, will provide us a novel therapeutic target to treat inflammatory or neuropathic pain releated to herpes virus infection. As summarized in Table 1, different subtypes of voltage-gated Na^+^ channels are involved in the development of inflammatory or neuropathic pain, and some of them have been reported to be associatiated with herpesvirus infecion-induced pain deveopment.

**Table 1:** Summary of voltage-gated sodium channel distribution and the related channelopathy

Channel	Main distribution	Inflammatory pain	Neuropathic pain	Herpes virus-induced pain	Other related diseases
Nav1.1	Large diameter DRG, myelinated A-fibers, inhibitory neurons	N/A	Increase [42, 52, 75]	N/A	Epilepsies, hemiplegic migraine and autistic spectrum
Nav1.2	CNS (dendrites and axons), excitatory neurons	N/A	Increase [75]	N/A	Epilepsies, intellectual disability and autism
Nav1.3	Fetal and neonatal DRG neurons, C-fibers	Increase [62, 66–68]	Increase [52, 66, 69–74] No change [13, 75, 76]	No change (lytic and latent) [13, 122]	N/A
Nav1.4	Skeletal muscle	N/A	N/A	N/A	Muscle paralysis episodes, myotonia episodes, infants apnoea, hypoxia, cyanosis or SIDS
Nav1.5	Cardiac tissue	N/A	N/A	N/A	Cardiac arrhythmias
Nav1.6	Large diameter DRG, myelinated A-fibers (Node of Ranvier)	Increase [90, 91] No change [86, 88]	Increase [75, 90, 91]	Increase (latent) [122]	EIEE, epilepsy
Nav1.7	Small diameter DRG	Increase [62, 102–105]	Increase [52, 66, 74, 107, 108] Decrease [37, 73]	Increase (latent) [13, 122]	Itch, IEM and PEPD (severe burning pain in rectum, eye and mandible)
Nav1.8	Small diameter sensory neurons	Increase [62, 66–68, 111, 112, 115]	Increase [52, 66, 74, 113–115] Decrease [37, 73, 106]	No change (Latent) [122]	MS and cardiac arrhythmias
Nav1.9	Small diameter DRG	Increase [52, 66, 68, 74, 118–121] Decrease [37] No change [62, 67]	No change [118–120]	N/A	Itch

A report showed that HSV-1 infection caused a significant reduction in the expression of VGSC [10, 17], but it did not indicate the regulatory effect of specific subtypes

*N/A* not applicable

### Nav1.1

Nav1.1 is mainly expressed in large-diameter neurons in DRGs and there is detectable amount of Nav1.1 in small diameter neurons according to in situ hybridization results [43]. Since myelinated A-fibers are mostly Nav1.1-positive cells [44], this indicates that Nav1.1 is dominantly expressed in fast-spiking GABAergic neurons, such as basket cells and Purkinje cells [45–47]. Therefore, loss-of-function mutations of Nav1.1 in patients will inactivate the inhibitory neurons and induce sever epilepsies [48, 49]. Mutations in Nav1.1 have been also reported in many other clinical diseases, including hemiplegic migraine and autistic spectrum [47, 49, 50]. Interestingly, Nav1.1 mutations are rarely reported in pain signaling [51]. However, a couple studies revealed that peripheral nerve injury induces an increase in Nav1.1 protein synthesis, which indicated that Nav1.1 may participate in nerve injury induced neuropathic pain [42, 52]. Therefore, Nav1.1 may play a role in pain signaling.

### Nav1.2

Nav1.2 is predominantly expressed in the central nervous system, but its expression in DRG is low. Nav1.2 accumulates on dendrites and pre-myelinated/unmyelinated axons according to in situ hybridization and immunohistochemistry [53]. Nav1.2 is important for early intellectual development, and de novo mutations in Nav1.2 channels are frequently reported to cause severe epilepsies, intellectual disability and autism [54–57]. Nav1.2 loss-of-function mutations decreases backpropagation of action potentials into cortical neurons through dendrites, which prevents synaptic plasticity, resulting in autism and intellectual disability [58, 59].

Unlike Nav1.1, which is mainly expressed in inhibitory neurons, Nav1.2 is dominantly expressed in excitatory neurons. Therefore, Nav1.2 is most likely to induce epilepsies though gain-of-function mutations [59, 60]. However, loss-of-function mutation of Nav1.2 also contributes to epileptic seizures, which could be due to the reduced excitability of Nav1.2-positive inhibitory neurons or the impairment of excitation/inhibition balance in Nav1.2-positive excitatory neurons [60, 61]. Changes in Nav1.2 mRNA expression during peripheral nerve injury and inflammation are rarely detected, indicating that Nav1.2 may have a minor effect on inflammatory and neuropathic pain development [62, 63].

### Nav1.3

Nav1.3 is abundant in fetal and neonatal DRG neurons, but rare in healthy adult DRG neurons. During embryonic development, the functional expression of Nav1.3 in the neocortex is not correlated with cell excitability, but regulates intracellular Ca^2+^ concentration required for synapses formation [64, 65]. In adult neurons, functional expression of Nav1.3 regulates the neuronal excitability.

Nav1.3 and Nav1.8 accumulates in uninjured neurons following nerve injury and they colocalizes with TNF-α [66]. Blocking TNF-α expression dramatically decreases Nav1.3 and Nav1.8 expression, which indicates that Nav1.3 and Nav1.8 may play an important role in inflammatory and neuropathic pain development [66]. Peripheral injection of complete Freund’s adjuvant (CFA) and carrageenan increases the mRNA and protein expression of Nav1.3, Nav1.7, Nav1.8 and Nav1.9 in DRG neurons [62, 67, 68], indicating that these sodium channels regulates the inflammatory pain.

Nav1.3 upregulation was reported during peripheral nerve injury, such as spinal nerve ligation, sciatic nerve transection or chronic constriction [69–72]. In chronic constriction injury of trigeminal ganglia neurons, only Nav1.3 is upregulated at both the mRNA and protein levels, indicating a pivotal role of Nav1.3 in developing trigeminal neuralgia (TN) [73]. Dysregulation of voltage-gated Na^+^ channels causes spontaneous neural activity and ectopic discharges, which are thought to be important for neuropathic pain development. However, Nav1.3 is mainly expressed in C-fibers that are rarely firing spontaneously [38], suggesting that other subtypes may cooperate with Nav1.3 for ectopic discharges. Consistent with this idea, expression of Nav1.3 was reported to be increased together with Nav1.7, Nav1.8 and Nav1.9 during nerve injury [52, 66, 74]. However, Nav1.3 expression does not change in a model of experimental paclitaxel-induced neuropathic pain, though there is a significant increase in the expression of Nav1.1, Nav1.2 and Nav1.6 [75]. A similar regulatory profile was also shown in a model of virus-induced neuropathic pain, in which Nav1.7 was upregulated during HSV-1 latent infection, while there was no change in Nav1.3 expression [13]. Furthermore, mice lacking the expression of Nav1.3 could still develop allodynia and produce ectopic discharges following nerve injury [76].

These controversial findings suggest that expression of Nav1.3 during induction of neuropathic pain may be affected by the specific localization of nerve injury. Thus, peripheral ventral root neurite injury but not the central axonal projection (dorsal rhizotomy) injury could upregulate Nav1.3 expression [66, 69]. Viral infection may cause injury of both peripheral and central nerves; therefore, the overall neuronal response to viral infection induced injury does not really change the expression of Nav1.3 channels.

### Nav1.4 and Nav1.5

Unlike other voltage-gated Na^+^ channels that are mainly expressed in the central nervous system (CNS) or peripheral nervous system (PNS), Nav1.4 is primarily expressed in skeletal muscle and Nav1.5 is predominantly in cardiac tissues [41]. Gain or loss-of-function mutations of Nav1.4 and Nav1.5 channels can result in muscular and cardiac disorders, respectively.

Nav1.4 is a TTX-sensitive sodium channel. As a muscle action potential initiator, its mutation-induced channelopathies directly affect the skeletal muscle excitability, resulting in episodes of muscle paralysis episodes or myotonia [77, 78]. Muscular hyperexcitability, resulting from gain-of-function mutations of Nav1.4, may trigger paramyotonia congenita or potassium aggravated myotonia. Loss-of-function mutations of Nav1.4 induce muscular hypoexcitability, resulting in hyperkalemia periodic paralysis [77]. Clinically, Nav1.4 gain-of-function variants affect the respiratory muscle of infants, leading to apnoea, hypoxia, cyanosis, or even sometimes sudden infant death syndrome (SIDS) [79–81]. Due to the specific tissue localization of Nav1.4, its dysfunction has never been reported to be associated with neuropathic pain disorders.

TTX-resistant Nav1.5 channel, which is predominantly expressed in cardiac muscles, is not involved in pain signaling but can trigger cardiac arrhythmias [82, 83]. Prolonged Q-T interval in the ventricular action potential was reported to be associated with Nav1.5 gain-of-function mutations [83]. Nav1.5 loss-of-function mutations are associated with inhomogeneous electrical conduction, that can lead to ventricular arrhythmias [83]. Interestingly, Nav1.5 is also expressed in colon cancer cells, and their activity regulates the transcription of cancer invasion-related genes [84].

### Nav1.6

Similar to Nav1.1, Nav1.6 is predominantly expressed in myelinated A-fibers. It is specifically located in the Nodes of Ranvier, which facilitates the rapid conduction of nerve impulses along the axon [85, 86]. Nav1.6 gain-of-function mutations in patient can lead to early infantile epileptic encephalopathy (EIEE), or even sudden unexpected death in epilepsy [87]. Nav1.6 contributes to 60% of the TTX-sensitive sodium current in large diameter DRG neurons, and it accumulates after nerve injury [86].

Tissue damage induced inflammatory pain does not change Nav1.6 expression at either the mRNA or protein levels, which indicate that Nav1.6 may have no effect on inflammatory pain [88]. Nav1.6 is rarely reported in pain development, but Nav1.7, Nav1.8 and Nav1.9 have been widely studied in different pain models [86]. Nevertheless, the regulatory effect of Nav1.6 on pain signaling transduction was reported to be masked by Nav1.8, since lack of Nav1.6 in Nav1.8-positive neurons showed no influence on acute inflammatory or neuropathic pain behaviors, while in Nav1.8-negative large DRG neurons, significantly ameliorated spared nerve injury induced allodynia [86].

Sympathetic sprouting into DRG functionally increase spontaneous activity of myelinated sensory neurons in several pain models. Local knockdown of Nav1.6 with siRNA in DRG before spinal nerve ligation (SNL) strongly reduces mechanical pain by blocking abnormal spontaneous firing activity [89]. Inflammatory cytokine TNF-α upregulates the expression of Nav1.6 or facilitates Nav1.6 trafficking to the membrane to increase the neuronal excitability, promoting the development of both inflammatory and neuropathic pain [90, 91]. Although, the effect of Nav1.6 on neuropathic pain development was rarely reported, several studies suggested that Nav1.6 is a promising therapeutic target to block neuropathic pain transmission.

### Nav1.7

Nav1.7 is a TTX-sensitive sodium channel that is dominantly expressed in small sensory neurons [43, 92]. Its slow close-state inactivation kinetics disables its response to high-frequency stimulation but helps small stimuli amplification [93]. Therefore, Nav1.7 may serve as a subthreshold amplifier and activate other sodium channels, such as Nav1.8, to produce and propagate the action potentials [94]. Gain-of-function mutations in Nav1.7 may lead to paresthesia or painful conditions, such as itch, inherited erythromelalgia (IEM) and paroxysmal extreme pain disorder (PEPD) [95–97].

In small diameter DRG neurons, there is detectable expression level of Nav1.6, Nav1.7, Nav1.8 and Nav1.9 in nerve terminals [98], which indicates that these channels may regulate the generation of presynaptic potentials and mediate the synaptic neurotransmission and nociceptive signaling [99–101]. Nociceptive transmission is mainly mediated by Nav1.7 channel activation in afferent neurons. Therefore, noxious stimuli or tissue damage induced nociceptive pain or inflammatory pain is inwardly transmitted by Nav1.7 mediated signaling. Indeed, the functional expression of Nav1.7 is significantly increased in both acute and chronic inflammatory pain models, indicating the critical role of Nav1.7 in inflammatory pain transmission [62, 102, 103]. Knockout or mutations of Nav1.7 strongly decreases the induction of hyperalgesia or even leads to the absence of pain sensation [104, 105].

The pivotal role of Nav1.7 in acute and inflammatory pain signaling has been widely studied, but its roles in neuropathic pain disorders is complex and requires further studies. In contrast to the upregulation of Nav1.7 in inflammatory pain, many reports demonstrate a significant downregulation of Nav1.7 expression in the development of neuropathic pain [37, 73, 106], indicating that the presence of Nav1.7 is not necessary for neuropathic pain development. Nav1.7 plays a pivotal role in inflammatory pain behaviors in mice, while it has no effect on neuropathic pain disorders [106]. In the rat sciatic nerve crush injury and spared nerve injury models, there is increased expression of Nav1.3, whereas the expression of Nav1.7, Nav1.8 and Nav1.9 decreases significantly [37]. Consistent with this observation, infraorbital nerve-chronic constriction injury induces trigeminal neuralgia and decreases Nav1.7, Nav1.8 and Nav1.9 mRNA and protein levels [73]. However, some other studies have demonstrated increased expression of Nav1.7 during nerve injury, indicating that Nav1.7 plays a critical role in the development of neuropathic pain as well [107, 108]. Cancer-related neuropathic pain has been reported to be associated with chemotherapy-induced peripheral neuropathy (CIPN) following intravenous administration of paclitaxel in cancer patients [107, 108]. Moreover, decreased ubiquitin ligase NEDD4-2 expression in injured DRG results in enhanced Nav1.7 and Nav1.8 currents and redistribution of Nav1.7 channel towards peripheral axons, which contribute to the development of neuropathic pain [108].

### Nav1.8

Nav1.8 is widely expressed in small diameter sensory neurons. As a TTX-resistant sodium channel, it mediates the slow activation and slow inactivation of sodium currents and it is physiologically coupled with Nav1.7 [88]. Therefore, many researchers studied Nav1.7 and Nav1.8 channels concomitantly. Nav1.7 and Nav1.8 are differently expressed in human DGR neurons and mouse DRG neurons. PCR analysis indicates that Nav1.7 participates in around 50% of the total sodium currents, and Nav1.8 constitutes 12% in human DRG neurons; while in mouse, there is 45% of Nav1.8 and 18% of Nav1.7 expression [109], which indicates that in vivo studies in mice or rats may be partially applicable in human. Ectopical expression of Nav1.8 was reported in multiple sclerosis (MS) and arrhymia [110].

The axon terminal expression of Nav1.8 indicates its function on nociceptive transmission and inflammatory pain [98]. In the complete Freund’s adjuvant (CFA) administration induced inflammatory pain model, there is increased Nav1.8 current density and reduced threshold for action potentials. However, blocking Nav1.8 sodium current reduces the CFA-induced allodynia [111]. This study indicates the critical effect of Nav1.8 on inflammatory pain development. In addition, a significant increase in the functional expression of Nav1.8 was detected in both myelinated and unmyelinated axons in afferent fibers following inflammation induced peripheral sensitization [112].

As with Nav1.7, the downregulation of Nav1.8 mRNA during nerve injury may indicates that Nav1.8 expression may not contribute to the genesis of neuropathic pain [37, 73, 106]. Surprisingly, in a spinal nerve ligation (SNL) induced pain model, Nav1.8 expression is increased and redistributed in unmyelinated axons of uninjured neurons [113]. Moreover, TNF-α was reported to induce Nav1.8 upregulation in uninjured sciatic nerve fibers following L5 ventral root transection (L5-VRT) [66]. Inhibition of TNF-α synthesis blocks the upregulation of Nav1.8 and prevents the development of neuropathic pain [66]. Furthermore, the intrathecal (IT) administration of Nav1.8 antisense oligodeoxynucleotide (ODN) selectively decreased Nav1.8 protein level and reversed neuropathic pain after L5/L6 spinal nerve ligation (SNL) [114]. Nav1.8, just like Nav1.7, plays a vital role in the development of inflammatory pain. In neuropathic pain disorders, the upregulation or redistribution of this channel in uninjured nerve ending could be the main mechanism for enhanced pain signaling. Daou’s study emphasized the essential role of Nav1.8 in both inflammatory and neuropathic pain disorders by using a novel transgenic mouse model, in which Nav1.8-positive afferent terminals can be optogenetically silenced with high spatiotemporal precision, resulting in alleviated inflammatory and neuropathic pain [115].

### Nav1.9

Nav1.9 generates TTX-resistant sodium channel. It is found in small diameter DRG neurons and exclusively expressed in nociceptive sensory neurons [88, 92]. Nav1.9 activation potential is close to the resting membrane potential. Therefore, it can be activated by subthreshold stimuli [116], which may contribute to the initiation of action potential. Notedly, persistent excitability conditions result in the inactivation of most of this channel [92], indicating that Nav1.9 might not contribute to neuropathic pain genesis. A recent report indicated that Nav1.9 plays an important role in acute itch [117].

Similar to Nav1.7 and Nav1.8, Nav1.9 was found in the nociceptive nerve terminals to mediate nociceptive signaling, which contributes to the development of inflammatory pain [98, 118, 119]. Nav1.9 knockout mice exhibit decreased thermal hypersensitivity induced by puerperal inflammation but not in neuropathic pain models [118–120], which indicates the crucial role of Nav1.9 in inflammation-induced hyperalgesia. A human Nav1.9 over-expressed ND7/23 cell model was used to investigate the role of Nav1.9 in modulating inflammatory pain [121]. In this model, gain-of-function mutations of human Nav1.9 showed significantly increased Nav1.9 activity, and the inflammatory mediator, histamine, enhanced the Nav1.9 activity [121], which linked Nav1.9 abnormal expression to inflammatory pain.

In contrast to these studies, Nav1.9 activity is not required in other inflammatory pain models. In carrageenan-induced pain models, increased expression of Nav1.7 and Nav1.8, rather than Nav1.9, was detected [62, 67]. Nav1.9 expression also showed no differences in lingual nerve neuromas patients with or without neuropathic pain, while Nav1.8 was proved to be the primary contributor to pain sensation [118–120]. Nav1.9 could be a potential target for the treatment of inflammatory pain, but there is no evidence demonstrating its effect on neuropathic pain conditions yet.

### T-type Ca^2+^ channel and pain

T-type Ca^2+^ channels are low voltage activated calcium channel, which can be activated by membrane depolarization near resting membrane potentials. Activation of T-type Ca^2+^ channels leads to the influx of Ca^2+^ ion and thus increases the intracellular calcium concentration, which could promote calcium-dependent enzyme activation, gene transcription, neurotransmitter release and muscle contraction [123, 124].

These channels are widely expressed in the central and peripheral nervous systems, endocrine system, cardiovascular system and skeletal muscle system [124, 125]. Therefore, the abnormal expression, mutations or dysfunction of these channels could lead to various diseases, such as epilepsy, Parkinson’s disease, hypertension and pain [14, 16, 124, 126]. A recent report summarized that T-type Ca^2+^ channels are also widely expressed in various cancers, such as breast, glioblastoma, prostate, colon and ovarian cancers, which may regulate cancer cell proliferation, differentiation, survival and migration [127]. Many studies demonstrated that inhibition of T-type Ca^2+^ channel function could cause cytostatic effect and limit cancer progression [128–130]. T-type Ca^2+^ channels are important for boosting the action potentials by serving as a preamplifier for sodium spikes. Although voltage-gated Na^+^ channels are the main contributor in pain signaling, the effect of T-type Ca^2+^ channels in propagation of pain signal is still indispensable and thus should be highlighted here.

Three distinct α subunits of T-type Ca^2+^ channels, termed Cav3.1, Cav3.2, and Cav3.3, are encoded through three distinct genes (CACNA1G, CACNA1H, CACNA1I). These three subunits posses different electrophysiological and pharmacological properties, but all generate transient calcium currents with a variety of inactivation kinetics [131, 132]. Different alternative splicing variants results in different properties or functions in specific circumstances, such as different cell surface expression, permeation, activation and inactivation kinetics in tumor growth, development of epilepsy, cardiac hypertrophy and non-functional channels [133]. Therefore, their roles in neuronal excitability are tissue-specific and even cell-specific. As summarized in Table 2, different subtypes of T-type Ca^2+^ channels contribute to the development of inflammatory or neuropathic pain, and some of them have been reported to be associatiated with herpesvirus infecion-induced pain deveopment.

**Table 2:** Summary of T-type Ca^2+^ channel distribution and the related channelopathy

Channel	Main distribution	Inflammatory pain	Neuropathic pain	Herpes virus-induced pain	Other related disease
Cav3.1	Excitatory neurons, inhibitory GABAergic neurons, TG and DRG	Decrease [140, 141]	Increase [138, 139]	N/A	Absence seizure, sleep/weak activity
Cav3.2	Small and medium diameter DRG	Increase [146]	Increase [147–152]	Decrease (lytic) [12, 18]	Cardiovascular disease, autism, hyperaldosteronism and seizure
Cav3.3	Small diameter neurons, smooth muscle cells	Increased [146]	Increase [147, 148]	N/A	Absence seizure

A report showed that HSV-1 infection has no regulatory effect on the expression of VGCC [10], but it did not indicate the regulatory effect of specific subtypes

*N/A* not applicable

### Cav3.1

T-type Ca^2+^ channels are important in regulating neuronal excitability. Cav3.1 subunits were reported to be important in weight maintenance and sleep/wake activity, which indicated that Cav3.1 could be a potential anti-obesity target [134]. In another report, knockdown of Cav3.1 or its inhibition significantly decreased neuronal excitability in the ventral tegmental area, as a result of decreased T-type currents and rebound burst firing in mouse or rat neurons [135]. Alpha(1G)-deficient mice are resistant to absence seizure due to the lack of burst firing in thalamocortical relay neurons [136], while Cav3.1 overexpression was reported to induce absence seizure [137].

Neuronal excitability is also related to transmission of pain signaling. Evidence showed that Cav3.1 plays an important role in regulating the development of trigeminal neuropathic pain [138]. In an infraorbital nerve ligation pain model, wild-type mice showed mechanical hypersensitivity and increased low-frequency rhythms compared with Cav1.3 knockout mice [139], indicating that these mice had increased pain sensation. Selective modulation of Cav3.1 subunit in T-type Ca^2+^ channels provided a novel gene therapeutic strategy to treat allodynia and hyperalgesia, since decreased response to mechanical stimulation and attenuated thermal hyperalgesia were observed in Cav3.1 null mice [139]. Cav3.1 is not only found in excitatory neurons, but also highly expressed in inhibitory GABAergic fast-spiking neurons in the periaqueductal gray (PAG). The crucial role of Cav3.1 in opioidergic descending analgesia was supported by the evidence that mutant mice (alpha 1G(−/−)) lacking low-threshold spikes showed impaired morphine analgesia [140]. Consistent with this idea, increased visceral pain and persistent hyperalgesia were observed in Cav3.1 deficient mice, which indicates an antinociceptive mechanism of T-type Ca^2+^ channels [141].

### Cav3.2

According to in situ hybridization analysis, Ca3.2 subunits are more abundant in small and medium diameter neurons, especially in DRG neurons [142]. Transcriptional profile of cutaneous nerve ending or primary sensory neurons by using RNA-sequencing technology indicated that Cav3.2 and Cav3.3 subunits are selectively expressed in primary sensory neurons and mediate synaptic release [143, 144]. Trigeminal ganglion neurons or dorsal root ganglion neurons are bipolar nerve cells. Their peripheral nerve ending expresses various nociceptors, which could process the stress or damage and transfer the pain signals to their somas located in TG or DRG. Their central nerve endings transmit the pain signals to higher central neurons, generating the order to avoid the damage.

Cav3.2, as an important pain signaling mediator, is predominately expressed in the synapses to mediate neurotransmitter release. It is also expressed in the soma to facilitate the action potential production and propagation. Many studies have been performed to support the major role of Cav3.2 in nociceptive, inflammatory pain and neuropathic pain [145–148]. De-inhibition of T-type Ca^2+^ channel Cav3.2 significantly increases the “Cav3.2-like” T-type current and lowers the threshold for nociceptor activation [145]. The upregulation of Cav3.2 and Cav3.3 expression contributes to the development of thermal hyperalgesia and tactile allodynia in chronic compression of dorsal root ganglion (CCD) pain model in rats [146]. Chronic constrictive injury of sciatic nerve induced neuropathic pain showed a significant upregulation of T-type channel Cav3.2 subunits, which highly increased the action potential firing probability [147]. In addition, nerve injury promotes the increase of T-type currents and upregulation of Cav3.2 and Cav3.3 mRNA [148]. The inhibition of T-type channels normalizes the painful behavior induced by nerve injury [148].

Nerve injury is certainly the main etiology for neuropathic pain. However, some other neuropathic disorders, such as diabetic neuropathies, toxic neuropathies as well as herpetic neuropathies, also contribute to the development of neuropathic pain [149–152]. These studies all showed T-type channel Cav3.2 subunits play a critical role in disease, toxin or virus-related neuropathic pain. Cav3.2 activity is also important for cardiovascular disease, since inhibition of Cav3.2 showed anti-hypertensive and anti-antiarrhythmic effects [153, 154]. Gene mutations of Cav3.2 channels were reported to be associated with autism, hyperaldosteronism and seizure [155–157]. Gene therapy toward these mutant Cav3.2 T-type channels could be one of the strategies to treat Cav3.2 mutation-related disease.

### Cav3.3

Cav3.3 knock out mice showed absence of burst firing and increased susceptibility to drug-induced absence seizures [158]. Contrary to the effect of Cav3.2 on the cardiovascular system, Cav3.3 cooperats with Cav1.2 to regulate arterial tone through mediating smooth muscle contraction in human cerebral arteries [159]. Cav3.3 was rarely studied in pain behavior separately. Upregulation of both Cav3.2 and Cav3.3 expression has been demonstrated in hyperalgesia and allodynia pain models [146–148]. Cav3.3 is usually co-expressed with Cav3.2 in small diameter neurons. To some extent, Cav3.3 may facilitate Cav3.2 functioning. Therefore, it will be interesting to test the potential synergistic effect of Cav3.3 and Cav3.2 in the near future. In addition, whether gain-of-function mutations of Cav3.3 or Cav3.3-deficiency plays a role in neuropathic pain progression is also intriguing for future studies.

## Therapeutic strategies towards HSV-1 induced pain

HSV-1 infection is associated with inflammatory or neuropathic pain. HSV-1 induced tissue damage, characterized by skin or mucosal epithelial cell lysis, triggers host immune defense and inflammatory infiltration. Inflammatory cytokines, chemokines or other mediators can induce pain response to tissue damage. For example, HSV-1 or PRV infection of rat embryonic fibroblast (REF) cells can induce cyclooxygenase-2 (COX-2) transcription and increase prostaglandin E_2_ production [160, 161], which is known to be involved in inflammation-induced pain.

Acute eye infection by HSV-1 or VZV induces corneal necrosis or retinal necrosis [162–166]. The ocular pain caused by herpes virus acute infection could be due to tissue damage-induced inflammatory pain. During viral infection, a variety of cytokines, like IL-4, IL-6, IL-8, IFN-γ, TNF-α and IL-1β, are released [167, 168]. Some of these cytokines such as TNF-α, IL-6, IL-1β were reported to increase neuronal excitability by upregulating the expression of voltage-gated ion channels. The effect of TNF-α on voltage-gated Na^+^ channels has been studied extensively. It was reported that TNF-α upregulates Nav1.3, Nav1.6 and Nav1.8 expression and trafficking during nerve injury. Inhibition of TNF-α synthesis prevents the increase of these sodium channels during nerve damage [66, 90, 91, 169]. IL-6 was reported to act as a neuroprotective cytokine and help axon regeneration after peripheral nerve injury [170]. IL-6 restores T-type Ca^2+^ channel functional expression post HSV-1 lytic infection [18]. The proinflammatory cytokine IL-1β was reported to induce pain sensation and has a bi-phasic effect on the regulation of voltage-gated Na^+^ channels in trigeminal nociceptive neurons [171]. Acute exposure to IL-1β reduces sodium currents, while chronic exposure increases sodium currents significantly [171]. Dynamic change of cytokine blood concentration during HSV-1 relapse and remission has been also observed clinically [172]. Bailey et al. reported that IL-2, IL-10, IFN-γ and TNF-α mRNA levels are elevated during HSV-1 latent infection in trigeminal ganglion even 4 months post-infection [173]. Therefore, ocular chronic pain could be the result of neuropathic pain, induced by the upregulation of some specific voltage-gated ion channels in response to increased cytokine release.

The host immune response towards HSV-1 acute infection helps in the conversion of lytic infection to latent infection, which promotes the retrograde transport of HSV-1 particles from the axon terminal to the neuronal perikaryon. This process can promote the establishment of viral latency in the trigeminal ganglia (TG) or dorsal root ganglia (DRG). Stress induced HSV-1 reactivation could lead to the infectious virions antegrade transport from the neuronal cell body to central nerve ending in the brain or peripheral nerve ending near other sensory neurons or tissues, causing both peripheral and central neuropathic disorders.

Neuropathic disorders induced by HSV-1 infection could result in neuropathic pain characterized by altered pain sensitization, including hyperalgesia and allodynia [9, 174, 175]. Many factors could induce pain sensitization, such as increased ion channel expression and excitatory neurotransmitter release, activation of pain signaling receptors, or decreased inhibitory neurons and inhibitory neurotransmitter release [20, 24]. Virus attack induced-apoptosis of neurons in the PNS and CNS could be another reason of neuronal sensitization [26].

Neuropathic pain is generally believed to be induced by changes in expression and function of nociceptors, ligand-gated or voltage-gated ion channels in sensory neurons, dorsal root ganglion (DRG) neurons and nociceptive afferent neurons [176, 177]. HSV-1 infection of sensory neurons was also reported to regulate the transmission of pain information by increasing or decreasing the neuronal excitability [10, 11]. Therefore, understanding the regulatory effect of HSV-1 on voltage-gated Na^+^ channels and T-type Ca^2+^ channels in sensory neurons will be helpful in developing effective druggable targets to treat HSV-1 induced herpetic pain.

### Therapeutic potential of targeting T-type Ca^2+^ channels

T-type Ca^2+^ channels draws increasing attention of researchers in regulating pain signaling since they open at subthreshold membrane potential. These channels have distinct biologic properties, including low activation threshold, relatively fast activation and inactivation kinetics, accumulation in the axon initial segment, and considerable expression in pre- and post-synaptic nerve terminals [178]. These properties enable T-type Ca^2+^ channels to control subthreshold neuronal excitability, neurotransmitter release as well as action potential generation. T-type Ca^2+^ channels can regulate pain signaling through many different mechanisms, such as decreasing the firing threshold, enhancing synaptic transmission, acting as mechanosensors, activating the ERK signaling pathway, or being in synergy with other ion channels [124].

Many reports have indicated that nerve injury induced neuropathic pain causes dysregulation of T-type Ca^2+^ channels [148]. Downregulation or deletion of T-type Ca^2+^ channels helps relieve nerve injury induced pain sensation [139, 141, 148, 179]. Interestingly, silencing of T-type channel Cav3.2 with antisense oligodeoxynucleotide resulted in antinociceptive, anti-hyperalgesic, and anti-allodynic effects in mononeuropathic rats [179]. In addition, Cav3.2 knockout mice displayed attenuated acute and chronic pain sensation [180]. Selective T-type Ca^2+^ channel blockers, like TTA-P2 and TTA-A2, could significantly decrease both inflammatory and neuropathic pain genesis [181, 182]. These inhibitors are currently being tested for clinical application to treat pain disorders.

HSV-1 infection induced neurodegeneration or neuronal cell death could also cause neuropathic pain. However, the regulatory effect of HSV-1 on T-type Ca^2+^ channels has not been well characterized. HSV-1 lytic infection reduces T-type Ca^2+^ and voltage-gated Na^+^ channel functional expression significantly [149–152], resulting in decreased neuronal excitability and disrupted pain signaling. There are two different outcomes when T-type Ca^2+^ channel expression decreases. First, in excitatory neuronal cells, decreased T-type Ca^2+^ channels lead to decreased pain sensation, which corresponds to HSV-1 induced hypoalgesia [138, 139, 145–148]. Second, in inhibitory neurons, decreased T-type Ca^2+^ channels may induce decreased inhibitory signaling to increase pain signaling, which corresponds to HSV-1 induced hyperalgesia or allodynia [140, 141].

Antiviral drugs inhibit viral replication and protein synthesis, which also prevent the downregulation of T-type Ca^2+^ channels [149–152]. However, viral drug-resistant mutations can make these antiviral drugs ineffective. For example, ACV is phosphorylated by viral thymidine kinase (TK). The ACV active form is deoxyguanosine triphosphate analog, which can compete to bind with DNA polymerase for DNA chain elongation [183]. DNA polymerase or viral thymidine kinase mutations result in no response to ACV or similar drugs [184–186]. Therefore, new therapeutic strategies, such as combination treatment or new drug discovery, are needed to be developed to avoid virus drug-resistant mutations. Furthermore, functional antiviral drugs may have no effect on relieving herpetic pain. Hence, targeting T-type Ca^2+^ channels during HSV-1 infection could be another efficient therapeutic method to treat herpetic pain.

### Therapeutic potential of targeting voltage-gated Na^+^ channels

As the main component of action potential generation, voltage-gated Na^+^ channels are involved in many inflammatory and neuropathic pain disorders [42, 52]. As with T-type Ca^2+^ channels, spontaneous activity, lowered thresholds, increased response to noxious stimuli or non-painful stimuli are the main characters of voltage-gated Na^+^ channel dysregulation on modifying the neuronal excitability. Inflammatory and neuropathic pain disorders correspond to differential alterations in voltage-gated Na^+^ channels, such as phosphorylation, trafficking and ectopic expression.

In mammals, ten different genes generate the pore-forming subunits of voltage-gated Na^+^ channels, and six of those, including Nav1.1, Nav1.3, Nav1.6, Nav1.7, Nav1.8 and Nav1.9, are involved in transmission of pain signals. Differential regulation of voltage-gated Na^+^ channels in various pain models makes these channels promising targets to treat specific pain disorders. According to etiology, Nav1.7 and Nav1.8 are the main channels responding to both neuropathic pains caused by neuropathy as well as inflammatory pains induced by tissue damage. Therefore, Nav1.7 and Nav1.8 are widely studied in many pain models [66–68, 106–108, 111–115] and haven been suggested as novel treatment targets. Neuropathic pain caused by various mechanisms, including spinal nerve ligation, sciatic nerve transection, chronic constriction and chemotherapy-induced peripheral neuropathy, can lead to the dysregulation of voltage-gated Na^+^ channels and result in the development of neuropathic pain. For example, in rat and human DRG neurons, paclitaxel-induced neuropathic pain showed enhanced Nav1.7 activity, increased Nav1.7 expression and spontaneous firing rate [107]. In spinal nerve ligation induced neuropathic pain disorders, Nav1.8 showed increased expression and ectopic distribution in axons, indicating an increased pain signaling in these models [113].

Virus-induced neuropathy, especially by viral latent infection, is another cause of neuropathic pain. VZV was reported to induce long-lasting back pain after skin heals, termed postherpetic neuralgia (PHN) [187, 188]. PHN VZV was reported to increase Nav1.6 and Nav1.7 expression compared to non-PHN VZV [122]. Although HSV-1 is usually not linked to postherpetic neuralgia (PHN), some clinical reports have indicated that HSV-1 plays a role in the development of long-lasting herpetic pain [30, 31]. It is important to investigate how HSV-1 regulates voltage-gated Na^+^ channels to understand HSV-1 induced persistent neuropathic pain. HSV-1 latency establishment in human DRG neuronal cells is a dynamic process with decreased and then increased neuronal excitability [13], which may explain the paralgesia caused by HSV-1 infection. HSV-1 latent infection induced upregulation of Nav1.7 expression but no change on Nav1.3 expression compared to non-infected neurons [13] indicated that Nav1.7 might be the main contributor to neuropathic pain and thus a potential target to treat postherpetic neuralgia. The full voltage-gated Na^+^ channel profile and the underlying mechanism of pain signals post viral infection are still not clear and need to be further characterized. TTX-sensitive sodium currents generated by the Nav1.1, Nav1.2 and Nav1.6 may also play a role in upregulating sodium currents in pain-transmitting neurons.

The increased pain sensation due to increased voltage-gated Na^+^ channel expression could be treated with channel toxins, antibodies, small molecular inhibitors or gene therapy [189]. Most of these treatments lack isoform or target selectivity, which can potentially induce various side-effects. For example, sodium channel blockers, such as lidocaine, tetracaine and phenytoin, induce lots of side-effects during local anesthesia due to their weak isoform selectivity [190, 191]. Therefore, discovery and development of novel small molecular inhibitors with high selectivity is a future research direction to treat neuropathic pain disorders. Some inhibitors with high selectivity, such as the clinical compound PF-05089771 and preclinical compound PF-05198007, specifically binds to the voltage-sensing region domain IV to inhibit Nav1.7 channel activity [192]. A-803467 and PF-01247324 showed more than 50-fold selectivity towards Nav1.8 over other sodium channels [193, 194]. ICA 121431, a Nav1.3 selective inhibitor, showed 1000-fold selectivity over other TTX-sensitive channels except Nav1.1 [195]. Although some of these compounds have been under clinical trial or in preclinical stage, their administration efficiency and long-term side-effects are still hard to predict. Therefore, more studies are needed to confirm the regulatory profiles of voltage-gated Na^+^ channels in different pain models, and more selective inhibitors are needed to be designed and investigated for potential clinical use.

## Closing remarks

Even after 100 years of studies, novel insights continue to pile up regarding this fascinating virus, particularly in the field of neuronal infection. Thanks to the novel genetic engineering tools, fluorescent-emitting viruses and new protocols of easy mutagenesis are providing very useful information toward the understanding of virus assembly, latency establishment, as well as reactivation. With the development of new human dorsal root ganglia neuronal culture and progress in high throughput electrophysiology, these new techniques are likely to offer noteworthy effect on future therapeutic treatments in encephalitis, keratitis, and neuropathy.

## Abbreviations

HSV-1: Herpes simplex virus type-1
VGSC: Voltage gated sodium channel
VGCC: Voltage gated calcium channel
DRG: Dorsal root ganglion
TG: Trigeminal ganglia
ACV: Acyclovir
TTX: Tetrodotoxin
HSE: Herpes simplex encephalitis
DAG: Diacylglycerol
NGF: Nerve growth factor
BDNF: Brain-derived neurotrophic factors
GDNF: Glial-derived neurotrophic factor
NT3: Neurotrophin-3
SIDS: Sudden infant death syndrome
EIEE: Infantile epileptic encephalopathy
IEM: Inherited erythromelalgia
PEPD: Paroxysmal extreme pain disorder
PAG: Periaqueductal gray
